# An Adapted Cancer Screening Education Program for Native American Women With Intellectual and Developmental Disabilities and Their Caregivers: Protocol for Feasibility and Acceptability Testing

**DOI:** 10.2196/37801

**Published:** 2023-02-13

**Authors:** Julie S Armin, Heather J Williamson, Janet Rothers, Michele S Lee, Julie A Baldwin

**Affiliations:** 1 Department of Family & Community Medicine The University of Arizona Tucson, AZ United States; 2 Center for Health Equity Research Northern Arizona University Flagstaff, AZ United States; 3 Department of Occupational Therapy Northern Arizona University Flagstaff, AZ United States; 4 Statistics Consulting Laboratory, BIO5 Institute The University of Arizona Tucson, AZ United States; 5 College of Nursing The University of Arizona Tucson, AZ United States; 6 Institute for Human Development Northern Arizona University Flagstaff, AZ United States; 7 Department of Health Sciences Northern Arizona University Flagstaff, AZ United States

**Keywords:** community-engaged research, community-engaged, engagement, cancer, breast, cervical, screening, breast cancer screening, cervical cancer screening, intellectual, developmental, cognitive, disability, disabilities, Native American, indigenous, women’s health, feasibility, acceptability, patient education, prevention, patient knowledge, health knowledge, health education

## Abstract

**Background:**

Women with intellectual and developmental disabilities (IDD) do not undergo breast and cervical cancer screening at the same rate as women without IDD. IDDs are diagnosed in childhood, are lifelong, and involve difficulties in adaptive behaviors and intellectual functioning. Native American women also experience disparities in breast and cervical cancer screenings. Despite known disparities, women with IDD are often not included in health promotion programs, and there is a need for evidence-based programming for those with intersectional identities, such as Native American women with IDD.

**Objective:**

This study aims to assess the feasibility and acceptability of My Health My Choice (MHMC), an adaptation of the Women Be Healthy 2 program. There are 2 parts to the study: adaptation of the Women Be Healthy 2 program and feasibility and acceptability testing of MHMC.

**Methods:**

Individuals aged over 18 years who identify as Native American females with IDD and their caregivers (N=30 women-caregiver dyads) are eligible for the study. Participants, who are affiliated with 2 partnering sites in Arizona (1 rural and 1 urban), complete pre- and postsurveys assessing knowledge, self-efficacy, and screening expectations before and immediately after completing the program. In addition, all participants complete brief satisfaction surveys after each of the 6 educational sessions. A subsample of Native American women with an IDD (n=12), caregivers (n=12), and community health educators (n=2) who participate in the MHMC program will provide semistructured qualitative input regarding the content, delivery, and cultural relevance of the program.

**Results:**

The adaptation of the culturally responsive MHMC program was completed in August 2021. In November 2021, the project team began recruitment for feasibility and acceptability studies. Feasibility will be examined using participation metrics, and acceptability will be measured using satisfaction measures. Pre- and postmeasures in cancer screening knowledge, self-efficacy, and screening expectations will examine improvements among participants.

**Conclusions:**

The results of feasibility and acceptability testing of MHMC will guide future implementation studies of the program.

**International Registered Report Identifier (IRRID):**

DERR1-10.2196/37801

## Introduction

### Background

About 1 in 4 adults in the United States report living with a disability [1]. Among US adults with disabilities, the prevalence of disability is higher among Native American populations and among young adults (aged 18-44 years), cognitive disabilities (including intellectual and developmental disabilities [IDD]) are the most prevalent [1]. IDDs are disabilities diagnosed in childhood that involve difficulty with adaptive behaviors and can include limitations in intellectual functioning [2].

### Cancer Screening in Women With Disabilities and Native American Women

Although screening rates for Native American women with IDD are not available, we do have data that indicate suboptimal screening in the intersectional population, especially regarding cervical cancer screening. Women with disabilities, particularly those with IDD, do not undergo cancer screening at the same rate as those without disabilities [3,4]. One reason that individuals with IDD do not receive cervical cancer screening may be that they have been perceived as asexual [5] and are thus less likely to receive comprehensive preventive sexual health care than women without IDD or other disabilities. For example, women with IDD are less likely to receive human papillomavirus screening compared with women without IDD [6]. While nearly 3 quarters of individuals with IDD (74%) reported having had a mammogram (vs 65.6% of women in the general population), only 57% of women with IDD reported cervical cancer screening in the past 3 years (vs 68.1% of women in the general population) [5-7]. Native American women are also less likely than the general population to undergo breast cancer screening (56.7% vs 71.5%, respectively) and cervical cancer screening (76.9% vs 83%, respectively) [8-10].

### Factors Influencing Adherence to Cancer Screening

The complex interplay among disability, culture, and health system-level factors complicates adherence to cancer screening. Many Native American people in the United States live in rural areas that present unique challenges to participating in preventive health care and require persistence to complete screenings [11,12]. Individuals with disabilities face cultural biases that affect the way health care professionals perceive them and may influence their preventive health care, including cancer screening [13,14]. Women with IDD and Native American women are at an increased risk of interpersonal violence [15-19]. In addition, individuals with IDD have experienced ableism and trauma in broader society and health care interactions [17], and those who are also Native American experience historical trauma [18]. Historical trauma refers to the violence imposed on Native American communities over multiple generations and includes US policies of forced relocation, family separation, and the prohibition of spiritual activities [20]. While violence and trauma arise from different histories, these individual and collective experiences may result in hesitance to engage with health care institutions [18]. We discuss our approach to address these experiences in the *Theoretical Frameworks* section.

Furthermore, family and support networks may influence adherence. Culturally responsive health education programs for people with IDD [21] and Native American communities [22] often involve families or other supporters. Women with IDD who live at home with their families are less likely to receive preventive health care services compared with those living in residential facilities [23]. Individuals from racial and ethnic minority groups, including Native Americans, are more likely to live with family caregivers compared with residential facilities [24]. Specifically, in Arizona, where this research is being conducted, 78% of individuals with IDD live with family caregivers [25]. Research indicates that caregivers (including family, friends, or paid support professionals) may believe that cancer screenings are unnecessary or that they do not want to make decisions for a person who cannot communicate their consent. Caregivers believe that by avoiding screening, they protect a woman with IDD from discomfort during screening or a possible cancer diagnosis [26]. Adding to the complexity of women’s self-advocacy, women with IDD living with family caregivers have less knowledge about breast and cervical cancer screenings compared with women with IDD living alone or with a spouse [27,28].

Efforts to develop educational resources specific for women with IDD to encourage breast and cervical cancer screening have demonstrated promising results [29-31]. *Women Be Healthy 2 (WBH2)* was developed to assist women with IDD who had expressed difficulties with or had never undergone breast or cervical cancer screening. After attending the *WBH2* program, participants showed improved knowledge about breast and cervical cancer screenings [31]. However, the *WBH2* curriculum was not implemented with Native American women; therefore, its relevance for this population is uncertain. In collaboration with community partners, this project aims to test the feasibility and acceptability of *My Health My Choice* (MHMC), an adaptation of the *WBH2* program for Native American women with IDD, to improve their cancer screening knowledge and self-efficacy.

### Original Intervention—WBH2

*WBH2* was selected for adaptation and testing, as a previous implementation of the program led to knowledge gains regarding breast and cervical cancer screenings among women with IDD [31]. *WBH2* consisted of 11 twice-a-week 60-minute sessions focused on the education about breast and cervical cancer screening, self-advocacy skill development, and techniques for anxiety reduction in women with IDD. The classes contained information on mammography or Papanicolaou testing, preparation for mammography or Papanicolaou testing procedures, breast and pelvic anatomy, and tools and skills for cancer screening visits. The course content was taught in an interactive way. For example, instructors read scenarios and asked students to consider what they would do if they were confronted with the challenges in the scenarios. Field trips to health providers to touch and see the equipment used in cancer screenings were also part of *WBH2*. *WBH2* did not proactively include caregivers as colearners in the program. *WBH2* was implemented and tested in a population of White and African American–identifying women with IDD attending urban adult day programs in a southeastern state in the United States [31]. Adult day programs are professionally run programs that adults with IDD attend to receive community living and other health-related programming [32].

### Theoretical Frameworks

#### Overview

This project is guided by the social model of disability [33] and socio-ecological model [34], which acknowledge the influence of political, social, and interpersonal structures on health. These frameworks allow the study team to complete a multilevel assessment of cancer screening disparities experienced by Native American women with IDD, which needs to be addressed in the content and structure of the education program. In the final refinement and testing of the culturally informed curriculum, we used the social cognitive theory (SCT) [35] in the design of our intervention, since it has been used previously by other researchers as a theory to affect cancer screening behavior change among Native American women [36]. SCT includes a construct, reciprocal determinism, which frames the interactions of women with IDD and influences their decisions to complete cancer screenings. This theory guides our data collection and analysis and is inclusive of other concepts, such as trauma-informed care and self-determination.

#### Trauma-Informed Approach and Self-determination

Within SCT, using a trauma-informed approach addresses internal and external reinforcements and their impact on health care decision-making. Trauma-informed approaches can also address the expectations construct in SCT, as women may have expectations about cancer screenings, based on previous trauma-inducing health care interactions. Trauma-informed approaches aim to prevent future retraumatization by providing a wide variety of delivery methods [37,38]. The program includes training for interventionists on trauma-informed methods and identifying potential behavioral triggers so that they are prepared to handle situations in which a program participant finds the course material emotionally difficult. The program also creates a safe space with ground rules for the discussion of topics.

Another important framing concept for this project is self-determination, which is central to both the Native American community and the IDD self-advocacy movement [39,40]. Self-determination among adults with IDD means making one’s own decisions regarding one’s needs and has been found to be a critical component for maintaining a good quality of life and for addressing population-level health disparities [41,42]. Among Native Americans, self-determination is necessary to reclaim ownership and control of community resources to meet the needs of the Native American population. Native American self-determination is needed for research on Native American populations [43]. This project promotes self-determination throughout the research process, as a community-engaged approach requires researchers and community members to work in equal partnerships through data collection, data analysis, and dissemination [44].

#### Objectives and Research Aims

The long-term goal of this community-engaged research project is to decrease breast and cervical cancer disparities among Native American women with IDD through culturally responsive and universally designed education in a southwestern US state. This paper provides a study protocol for an ongoing community-engaged research study focused on addressing breast and cervical cancer screening disparities among Native American women with IDD, via the delivery of a health education program titled MHMC. Here, we briefly describe how we used community-engaged methods to develop the MHMC program and then describe the feasibility and acceptability testing methods.

## Methods

### Project Timeline

**Figure 1:**
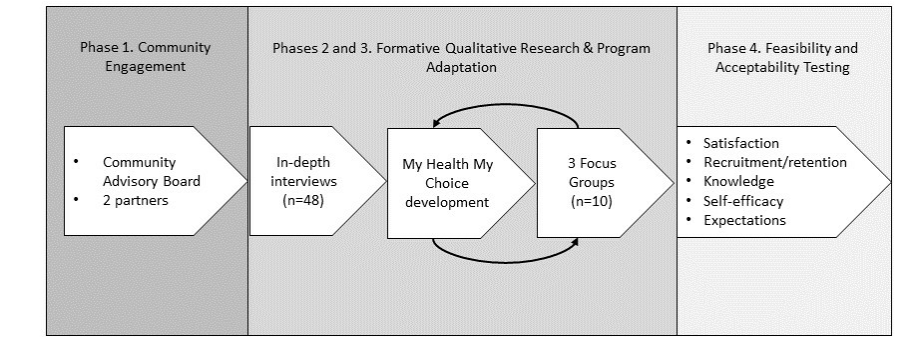
Study flow.

This project was initiated in September 2017 with the establishment of an advisory board and relationship-building with community partners in the research project. Once urban and rural community partnerships were established, the project team began 2 steps of formative qualitative research to inform the adaptation of *WBH2 (September 2019-August 2021)*. The project team began feasibility and acceptability testing of MHMC in November 2021. Figure 1 illustrates the study flow, including the phases of development, implementation, and testing of MHMC described in further detail below.

### Ethics Approval

The study was approved by the Institutional Review Board of the University of Arizona (STUDY00000034, September 28, 2021, with original protocol number 1709856263, approved September 27, 2017), with a ceded review by the Northern Arizona University Institutional Review Board (protocol #1132744-7). The study was also approved by all required tribally affiliated entities.

### Phase 1: Community Engagement

This study uses a community-engaged approach. Similar to Community-Based Participatory Research, community-engaged research is an equity-based approach that involves communities throughout the research process by considering important elements of the community (eg, resources and strengths that the community brings) [43,45,46]. This approach also consists of building trust with communities and involving community members throughout all phases of the project, including defining, implementing, and engaging in research. This project utilizes an advisory board design to serve as an element of community feedback and research oversight. This 11-person advisory board includes community members with familial ties to Native American women with IDD and representatives from cancer control programs, Native American health programs, and disability services. The advisory board has met quarterly for 1 to 2 hours since the inception of the project to provide ongoing supervision and feedback on the project, including ideas about how the program could be enhanced to meet community needs and suggestions for implementation. Early in the project, the advisory board was asked for input on initial revisions to *WBH2*, offering thoughts about the content and structure, including the need for cancer diagnosis information and for a reduction in the number of sessions [47]. A community-engaged approach was chosen for the project because of the growing recognition of the importance of including community members as partners in research efforts focused on addressing health [48,49].

### Phase 2: Formative Qualitative Research

Cultural adaptation of the *WBH2* curriculum in partnership with a Native American urban health and social services provider and a Native American rural cancer screening and support provider was complete at the time of this writing. Cultural adaptation occurs in 2 steps. In step one, individual in-depth interviews with Native American women with IDD, caregivers, health care and disability providers, and community members were completed. The results of the interviews [50] were discussed with the advisory board and members suggested program activities to address these findings. The results of the interviews provided an initial framework for culturally adapting the program, now called MHMC. Formative research and advisory board feedback on the findings led to changes in the program’s content and structure, including how and by whom it should be delivered. A total of 3 focus groups (n=10) were completed with both partner sites and throughout the state of Arizona to review and update the program. Preliminary program recommendations were reviewed via focus group discussions at each partner site with partner site program staff, caregivers, and Native American women with IDD to help the team refine the program.

Our community partners represent multiple tribal identities; one represents one tribe in the state, and the other is an urban Indian center that serves tribal members from all over the United States living in Southern Arizona. Acknowledging the diversity of tribal groups and the diversity among women with IDD allows the project to identify the diversity and commonality of experiences with breast and cervical cancer screening among Native American women with IDD.

### Phase 3: Adapting the Program (MHMC)

#### Overview

**Figure 2:**
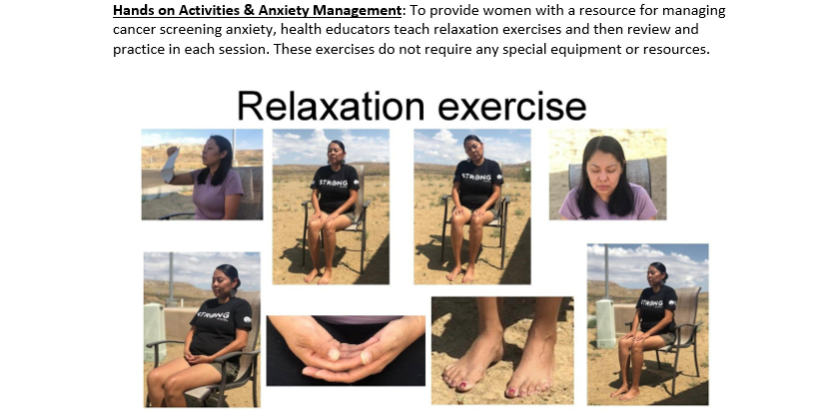
My Health My Choice (MHMC) program component.

**Figure 3:**
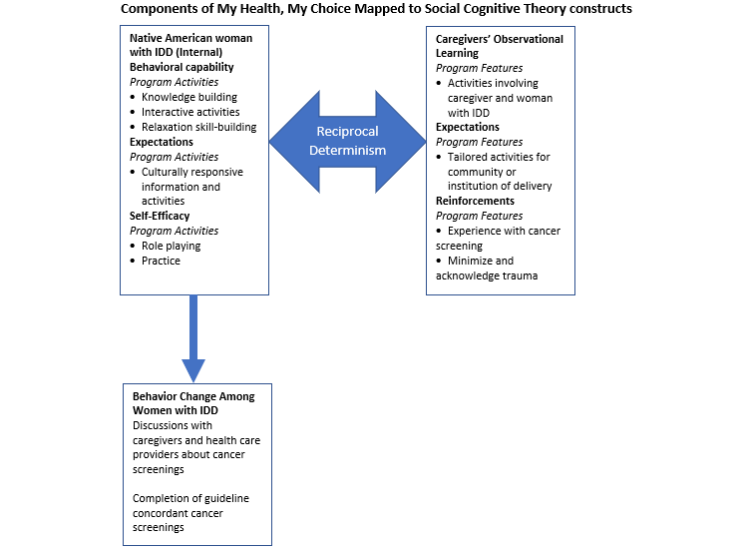
Components of My Health My Choice mapped to the social cognitive theory constructs. IDD: intellectual and developmental disabilities.

The adapted program takes place in six 1-hour sessions provided weekly over the course of 6 weeks. The education program addresses the personal, interpersonal, and environmental barriers to cancer screening experienced by Native American women with IDD. The program’s six learning objectives include (1) identify healthy lifestyle habits for women, (2) practice managing anxiety about cancer screenings, (3) identify what cancer is and the parts of the body being screened for cancer, (4) learn about breast cancer screening, (5) learn about cervical cancer screening, and (6) practice self-advocacy in health care. Figure 2 shows a screenshot of a program element. Key components include engaging Native American women with IDD and their care providers in discussions regarding cancer screening decisions, bringing in culturally relevant ways to share knowledge, and providing resources for dealing with the next steps in the case of screening results that require diagnostic testing. Below, we discuss how each of these components aligns with the constructs of the SCT. Finally, we describe how the COVID-19 pandemic generated additional curriculum delivery decisions. Figure 3 provides a summary of the SCT constructs aligned with program features.

#### Caregiver Involvement

Involving caregivers in the MHMC intervention addresses the central concept of SCT, reciprocal determinism, as women will make individual decisions regarding cancer screenings (internal) in collaboration with their caregivers, who can reinforce the decision.

#### Native American Cultural Elements

Native American cultural elements were also included in the program. The content highlights self-advocacy, using peers from the same Native American community to deliver the intervention, and ensuring that indigenous female healers rather than male healers are included. Culturally relevant imagery and metaphors are used to share the concepts of health, cancer, and self-advocacy. Providing culturally relevant materials may help address SCT constructs of reinforcement and expectations. Reinforcements are responses to behavioral choices that may influence future behaviors. Providing culturally relevant concepts to explain the importance of cancer screening may reinforce or encourage the decision to undergo cancer screening. Expectations are expected consequences of completing an activity. Demonstrating that participating in cancer screening is something that is expected and culturally accepted through culturally relevant materials may encourage screening.

#### Hands-on Activities and Anxiety Management

The use of hands-on activities and anxiety reduction techniques is provided with the intent to build knowledge, skills, and confidence through observational and experiential learning about cancer screenings among women with IDD and their caregivers. Within SCT, one must have self-efficacy (or confidence) as well as behavioral capability (knowledge and skills) to initiate a behavior. Observational learning in SCT refers to behavior change that can be encouraged through observations of modeled behavior. The program provides opportunities for hands-on activities to improve knowledge and understanding about a healthy body and cancer screening. The program includes tactile craft activities that demonstrate what will happen to a breast during a mammogram (ie, pressing a stress ball between your hands) and teaching about parts of the body involved in cancer screening (ie, creating a uterus and cervix with a baby sock). The stress ball activity can also help with anxiety in women with IDD about breast cancer screening as it shows pain visually (eg, showing a stress ball being squeezed in the mammogram machine) but also communicates how pain and discomfort pass. Each session opens and closes with relaxation technique exercises for women to practice managing their anxiety around cancer screening. Finally, women with IDD and their caregivers in the program also write out their plans and action steps to participate in cancer screening.

#### COVID-19 Modifications to Delivery of the MHMC Program

The plans for implementing the MHMC program were modified because of the COVID-19 pandemic. The project team modified the program to be delivered remotely to dyads of Native American women with IDD and their caregivers. Each participant has a MHMC toolkit delivered to their home with printouts of educational resources and items needed for hands-on activities. For women residing in urban areas with adequate internet access, the program is delivered via Zoom by a health educator. For women residing in more rural areas with limited or inconsistent internet access, the program is delivered over the phone by a health educator. A project website was developed to distribute educational materials. Videos were produced at each partner site using imagery and voice overs (ie, social story format) to walk women through the process of getting a Papanicolaou test and a mammogram with a local provider.

### Phase 4: Feasibility and Acceptability Testing

#### Overview

The study’s second aim, which is currently being completed, tests the feasibility and acceptability of MHMC and explores knowledge, self-efficacy, and screening expectation outcomes among Native American women with IDD and their caregivers*.* The culturally adapted MHMC program is implemented by female community health educators affiliated with our 2 community partners. Native American women with IDD (and their caregivers; N=30 student-caregiver dyads and n=15 dyads per partner site) complete pre- and postsurveys assessing knowledge, self-efficacy, and screening expectations before and immediately after completing the program. In addition, all participants complete brief satisfaction surveys following each of the 6 educational sessions (Multimedia Appendix 1). A subsample of Native American women with IDD (n=12), caregivers (n=12), and community health educators (n=2) who participated in the *My Health, My Choice* program provide semistructured qualitative input regarding the content, delivery, and cultural relevance of the program. Finally, the team tracks recruitment, attendance, demographic information, completion rates, and fidelity data for each session throughout the program.

The inclusion criteria for our second study aim are as follows: women with IDD must self-identify as Native American women with IDD and report being born a girl and caregivers must self-identify as caregivers for Native American women with IDD. Both types of participants must reside in Arizona, be aged over 18 years, and speak English or the other Native American language spoken in rural areas. Participating Native American women with IDD may choose a caregiver to participate in the program with them. The caregiver could be a family member, friend, legal guardian, or a paid support professional. If the caregiver identifies as a male, the questions collected at baseline and follow-up do not include information about their receipt of or intention to get screened for breast or cervical cancer. The following sections and Table 1 describe the measures and timing of the data collection (ie, T_0_ is time zero and T_1_ is time 1) used to evaluate the feasibility and acceptability of MHMC*.*

**Table 1:** Measures by participant type and intervention session time point.

All measures	Participant type	Time							
		T_0_	T_1_	T_2_	T_3_	T_4_	T_5_	T_6_	T_7_
Demographics	Woman with IDD^a^, caregiver	✓	—^b^	—	—	—	—	—	—
Cancer screening knowledge	Woman with IDD, caregiver	✓	—	—	—	—	—	—	✓
Cancer screening self-efficacy	Woman with IDD, caregiver	✓	—	—	—	—	—	—	✓
Cancer screening history	Woman with IDD, caregiver (female only)	✓	—	—	—	—	—	—	—
Cancer screening expectations	Woman with IDD, caregiver (female only)	✓	—	—	—	—	—	—	✓
Program satisfaction	Woman with IDD, caregiver	—	✓	✓	✓	✓	✓	✓	—

^a^IDD: intellectual and developmental disabilities.

^b^Data not collected at the time point.

#### Demographics and Screening History

We collect demographic information for each Native American woman with IDD (program participants) and their caregivers. Demographic information includes age, gender identity, disability status, race, ethnicity, level of education, employment status, health insurance coverage, home residence (ie, in one’s own home or group home), and guardianship status. In addition to demographic data, we collect information about the participant and female caregiver breast and cervical cancer screening history using questions from the PhenX toolkit [51]. Funded by the National Institutes of Health, the PhenX toolkit is a collection of standard data collection protocols for conducting biomedical research, thus allowing cross-study analysis [51].

#### Knowledge and Self-efficacy

We collect the following data from each MHMC participant at baseline and follow-up: (1) breast cancer screening knowledge, self-efficacy, and screening expectations (female participants only) and (2) cervical cancer screening knowledge, self-efficacy, and screening expectations (female participants only). Mammography knowledge is measured using the Mammography Preparedness Measure developed by Wang et al [52] to measure the understanding of mammography’s purpose and the procedure itself among women with IDD. Verbally administered, the instrument asks the participant to role-play, providing advice to the interviewer, who will be getting a mammogram. Questions are asked using plain language about the body parts checked by a mammogram, why it is used, how it is done, and how often it should happen. Parallel questions to assess cervical cancer screening or Papanicolaou testing knowledge along with scripts for both knowledge assessments were created by the authors of this study and are presented in Tables 2 and 3. We further adapted this instrument by including images of a mammography machine and an exam table with stirrups, as visual supports can assist individuals with IDD in processing information [53,54].

Self-efficacy refers to an individual’s perception of their capacity to perform certain tasks in their life [35]. To assess self-efficacy in breast and cervical cancer screening, we modified the colorectal cancer self-efficacy instrument [55] that has been used in a Native American population [56], as shown in Table 3. Participants view an image of a ladder and read a script that is loosely based on the self-anchoring striving scale by Cantril. [57]. The bottom of the ladder corresponds with a score of zero, or *not sure at all* and the top of the ladder corresponds with a score of 10 for *very sure*. Participants are asked the following question, “Imagine that this ladder is a way of picturing your confidence, or how sure you are that you can do something. The top of the ladder indicates that you are very sure, and the bottom indicates that you are not sure at all. For these next questions, what place on the ladder (or number between 0 and 10) matches how sure you feel?” Native American women with IDD and their female caregivers are then asked 4 questions regarding their ability to *decide* and to *get* a cancer screening (Tables 4 and 5). Both male and female caregivers are asked parallel self-efficacy questions regarding their confidence in *supporting* a woman with a disability that they care for through the cancer screening process.

**Table 2:** Breast and cervical cancer screening knowledge assessments script.^a^

Knowledge of breast cancer	Knowledge of cervical cancer
Script: *“A mammogram is a machine that looks for cancer in your body. Here is a picture of a mammogram machine. Today I am pretending to go to the doctor’s office and will be asked to get a mammogram.”* (Show image)	Script: *“Here is a picture of an exam table I would lay down on to get a Pap test*^b^*. Today I am pretending to go to the doctor’s office and will be asked to get a Pap test.”* (Show image)
“*Think back to a time that you went to the doctor’s office. Now, I want you to pretend that you are at the doctor’s office with me. I need your help in understanding what the doctor is asking me to do. Thank you for helping me.”*	“*Think back to a time that you went to the doctor’s office. Now, I want you to pretend that you are at the doctor’s office with me. I need your help in understanding what the doctor is asking me to do. Thank you for helping me.”*
“*Do your best. If you don’t know the answer it is okay.”*	“*Do your best. If you don’t know the answer it is okay.”*
“*Please make sure you help me on your own, do not have your family or others help you.”*	“*Please make sure you help me on your own, do not have your family or others help you.”*

^a^Questions regarding knowledge of breast cancer were adapted from the Mammography Preparedness Measure [52], and cervical cancer knowledge questions were modeled on these questions.

^b^Pap test: Papanicolaou.

**Table 3:** Breast and cervical cancer screening knowledge assessments.

Breast cancer questions	Answers considered correct	Cervical cancer questions	Answers considered correct
What part of the body is the mammogram for?	Breast (or any reasonable slang term)	What body part is a Pap^a^ test for?	Cervix, or inside the vagina, or private parts (or any reasonable slang term)
Why would I have a mammogram?	To check for cancer, must include cancer and screening (not curing, etc)	Why would I have a Pap test?	To check for cancer, must include cancer and screening (not curing, etc)
Would I have to take my clothes off for a mammogram?	Yes, top only, shirt and bra, etc	Would I have to take my clothes off for a Pap test?	Yes, bottom only, pants, underwear
When I have the mammogram how long will I be in the machine?	Any answer under 30 min	When I have my Pap test, how long will it take?	Any answer under 10 min.
How often am I supposed to have a mammogram?	Every year or every other year, annually, or based on when your doctor recommends	How often should I have a Pap test?	Every 5 years or based on when your doctor recommends

^a^Pap test: Papanicolaou test.

**Table 4:** Breast cancer and cervical cancer screening self-efficacy assessment script^a^.

Script	Assessment
*Script: imagine that this ladder is a way of picturing your confidence, or how* *sure* *you are that you can do something. The top of the ladder means that you are very sure, and the bottom means you are not sure at all. For these next questions, what place on the ladder (or number between 0 and 10) matches how sure you feel.”* ^b^	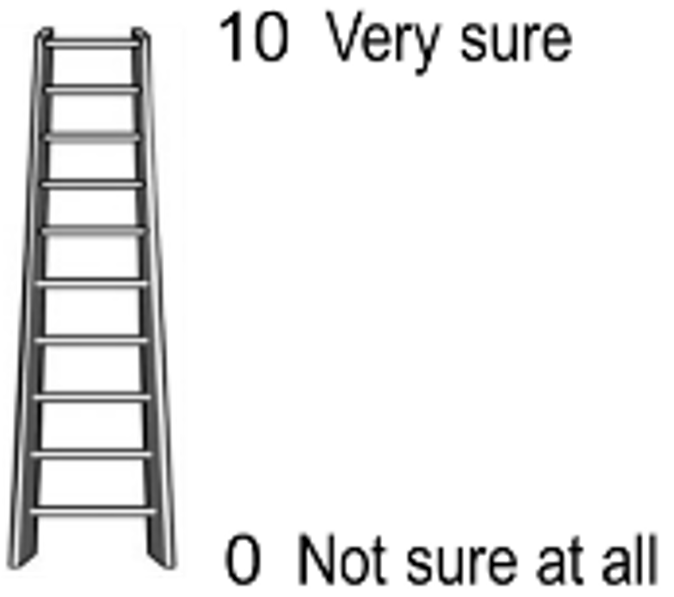

^a^Original instrument measuring self-efficacy in colorectal cancer screening was assessed using a four-point scale by McQueen et al [55] and further described in a Native American context by Frerichs et al [56].

^b^Adapted from self-anchoring striving scale by Cantril [57].

**Table 5:** Breast cancer and cervical cancer screening self-efficacy assessments.

Original question^a^	Breast or cervical cancer screening questions^b^	Caregiver modification^c^
How sure are you that you can decide whether or not to get screened for colon cancer?	How sure are you that you can *decide whether or not to get a mammogram/Pap*^d^ *test?*	...support the woman with a disability that you care for in deciding whether or not to get a mammogram/Pap test?
How sure are you that you can complete colon cancer screening?	How sure are you that you can *get a mammogram/Pap test?*	...support the woman with a disability that you care for to get a mammogram/Pap test?
How sure are you that you can complete colon cancer testing even if you are nervous about it?	How sure are you that you can *get a mammogram/Pap test, even if you are nervous about it?*	...support the woman with a disability that you care for to get a mammogram/Pap test, even if she is nervous about it?
How sure are you that you can do what is needed to prepare for colon cancer screening?	How sure are you that you can do what is needed to prepare for a mammogram/Pap test?	Not assessed

^a^The original survey had 8 questions, of which we use 4.

^b^Mammogram and Papanicolaou test asked as separate questions.

^c^Text in this column replaces underlined words from the previous column.

^d^Pap test: Papanicolaou.

#### Cancer Screening Expectations

Using the same ladder image described for self-efficacy questions, participants are asked to rank how they feel about planning to get a screening with zero, indicating that they are not planning to get a screening to 10 that they are definitely planning to participate in cancer screenings. Native American women with IDD and their female caregivers are asked 3 questions regarding their personal expectations to get cancer screening (Table 6). Both male and female caregivers are asked about their expectations in assisting the woman they care for to get their cancer screening (Table 6).

**Table 6:** Questions assessing participant and caregiver expectations for getting, or assisting with getting, a mammogram or Papanicolaou test^a^.

Breast or cervical cancer expectation questions^b^	Caregiver modification^c^
I plan to get a mammogram or Pap test^d^.	Assist the woman with a disability I care for in getting
I plan to discuss getting a mammogram or Pap test with my doctor.	Support the woman with a disability I care for to discuss
I plan to discuss getting a mammogram or Pap test with people who support me (for example: family member, friend, staff, helper, direct support professional).	Not assessed

^a^0 to 10 scale, shown via a ladder for self-efficacy questions in Table 4.

^b^Mammogram and Papanicolaou test asked as separate questions.

^c^Text in this column replaces underlined words from previous column.

^d^Pap test: Papanicolaou.

#### Acceptability

The acceptability of the program is captured by asking program participants to complete a brief satisfaction survey regarding the session’s format and content immediately after each session. Program feasibility is assessed by tracking the attendance and completion rates of all participants across all 6 sessions. A subsample of Native American women with IDD (12/30, 40%), caregivers (12/30, 40%), and community health educators (2/4, 50%) who participated in the *My Health, My Choice* program complete semistructured qualitative interviews regarding the content, delivery, and cultural relevance of the program. Fidelity is measured using data collected from Community Health Educators after each session. Data points include session start or end time, delivery method, challenges in delivery, ratings of the session, and participant engagement.

#### Analyses

Preliminary analyses of quantitative and qualitative data will be shared with the advisory board to assist with interpretation. On the basis of the advisory board input, we will finalize the analyses and determine the next steps for the research.

##### Statistical Analysis

Our primary outcomes will be participation metrics (feasibility and acceptability) including the percentage of women who choose to participate, the percentage of enrolled participants at each session, and the percentage of enrolled participants retained through the last session. A sample size of 30 Native American women with IDD and their caregivers from rural (15/30, 50% woman-caregiver dyads) and urban (15/30, 50% woman-caregiver dyads) settings will allow us to estimate participation and retention rates within an SE of 9% overall or within an SE of 13% within each setting (rural vs urban). Our secondary quantitative outcomes will be pretest to posttest differences for each of the following measures: (1) cancer screening knowledge and expectancy among Native American women with IDD; (2) self-efficacy scale for Native American women with IDD; (3) cancer screening knowledge and expectancy among caregivers; and (4) self-efficacy scale for caregivers. Assuming an attrition rate of 20%, a study with a sample size of 24 will provide 90% power to detect a standardized effect size of 0.66, that is, approximately two-third of SD in pre- and posttest scores. The 90% power estimate is conservative because it assumes the independence of the observations. However, when assessing these outcomes in the final analysis, we will address within-site (rural vs urban) and within-workshop session (1-6) correlation using mixed effects models. The proposed effect size of 0.66 is realistic based on a similar pre-poststudy conducted by Swaine et al [31], for which the effect size for composite score improvement was 0.83. For all other proposed comparisons, we believe that two-thirds of effect size represents a substantive improvement. We have not controlled for multiple comparisons in these analyses, but we note that the main purpose of this study was to be descriptive in nature, providing mean and variance estimates to be used for powering a future randomized controlled trial. All calculations herein assumed 2-sided tests with an α of .05.

Additional exploratory analysis will be conducted to assess relationships with variables that the investigators and previous research studies have identified as potentially influential on outcomes, such as participant residence (eg, group home, living independently, living with family, and urban or rural), the type of care provider attending the workshop (eg, paid and family), receipt of services from the state (eg, Medicaid, Indian Health Services, and Division of Developmental Disabilities), and the level of support needed. Specifically, we will examine whether changes in knowledge, attitudes, beliefs, and self-efficacy vary according to each of these variables. On the basis of previous studies, we anticipate that women who live in a group home or with the family will show greater knowledge and self-efficacy gains [26] and that women with greater support needs may show lower gains in knowledge and self-efficacy [31]. Because of the exploratory nature of this assessment, no adjustments will be made for multiple testing, and *P* values near .05 will be interpreted cautiously.

##### Qualitative Analysis

In-depth interviews will be transcribed and coded. Using methods for ensuring reliability among coders, transcriptions will be coded individually by separate members of the research team, who will then meet to analyze for agreement and reproducibility [58]. Coding will be completed in an iterative fashion, with initial (a priori) codes being drawn from the literature and additional codes based on themes that emerge from the data [59]. This modified grounded theory approach has been used by other qualitative researchers and proposed as a “best practice” to ensure content validity [60].

#### Advisory Board Involvement

Our advisory board will work with us to interpret quantitative and qualitative findings. The study team will present the findings at a quarterly advisory board meeting, inviting members who are interested in engaging in one-on-one discussions about the data with the university-based study team. Discussions with the advisory board will focus on understanding the data in light of the following topics: meeting community needs, opportunities for additional collaboration, and plans for further implementation.

## Results

This community-engaged research project began in September 2017. The study team developed a productive advisory board and established 2 community partnerships. Together, the university and community partners have completed a qualitative needs assessment to guide the development of a culturally responsive *My Health, My Choice* cancer screening education program for Native American women with IDD (September 2019–August 2021). The findings of in-depth semistructured interviews with Native American women with IDD (n=12), caregivers (n=11), disability and health care providers (n=20), and community members (n=2) in rural and urban contexts guided the adaptation of the program and have been reported in a separate manuscript [50]. Major adaptations to the content include information about integrating traditional and biomedical healing, and culturally relevant activities. Adaptations to the program structure include shortening its length and transforming it into remote delivery because of the COVID-19 pandemic. Importantly, female health educators are members of native communities. The MHMC program is provided by local community health educators using phone or video conferencing and web- or paper-based learning materials. The feasibility and acceptability testing of the MHMC educational program began in November 2021 with the goal of recruiting 30 dyads of Native American women with IDD and their respective caregivers. Measures were carefully selected based on their prior use and validation among Native Americans [55,56] or individuals with IDD [52].

## Discussion

### Overview

This paper describes a study protocol for a community-engaged and ongoing research project focused on addressing cancer screening disparities among Native American women with IDD. Given the paucity of research regarding health care decision-making among those living with intersectional identities (Native Americans with IDD) in the United States, a community-engaged approach is necessary. To date, the project team has built a community-relevant, trauma-informed, and universally designed cancer screening education program for Native American women with IDD. The significance of this study lies not only in its community-engaged approach, but also in its theoretical approach (SCT), which accounts for the multifactorial influences of trauma on screening behavior and incorporates them into the intervention.

### Principal Findings

We anticipate that the MHMC program will prove acceptable as assessed by a brief satisfaction survey, and feasible, as assessed by tracking attendance and completion rates of participants across all 6 sessions. We also expect to see an increase in cancer screening knowledge and self-efficacy across all participants.

### Strengths and Limitations

As noted in the introduction, the lack of screening adherence data for Native American women with IDD limits our ability to understand the broad significance of this study. However, throughout the project, the team presented at local community events and participated as educational vendors to stay connected with local community needs. As others have noted, community engagement can be helpful for establishing mutually productive and collaborative relationships with Native American communities [39]. Our approach to community engagement is a strength for understanding the health and health care experiences of a largely underrepresented group of individuals in health disparities research, Native American women with IDD. Given this study’s partnerships with specific Native American communities, the results of this work may not be generalizable to other groups of women with IDD from other Native American communities. However, these findings can provide a model for supporting Native American women with IDD in planning for cancer screenings, while considering the personal resources available to them.

### Dissemination Plans

In addition to the development and public availability of MHMC, the study team has published 3 manuscripts covering the state of research on cancer screening and treatment for individuals with IDD and the lessons learned from our community-engaged processes [45,61-62]. A fourth manuscript sharing detailed results of community-based qualitative formative research has been published [50], and the results of feasibility and acceptability testing will also be submitted for publication.

## Appendix

Multimedia Appendix 1 My Health My Choice Instruments.

## Abbreviations

IDD: intellectual and developmental disabilities
MHMC: My Health My Choice
SCT: social cognitive theory
WBH2: Women Be Healthy 2

## Data Availability

These data are not publicly available. Separate approvals must be obtained from tribal and community partners.
