# Supplementary material for: An Adapted Cancer Screening Education Program for Native American Women With Intellectual and Developmental Disabilities and Their Caregivers: Protocol for Feasibility and Acceptability Testing
Source: JMIR Res Protoc. 2023 Feb 13;12:e37801. doi: 10.2196/37801 (PMC9972207; doi:10.2196/37801)
Supplement: Multimedia Appendix 1 [file resprot_v12i1e37801_app1.docx]

**Screener Form Woman with IDD**

*Introduction*

Hello my name is ____. I am working on a project with the Partnership for Native American Cancer Prevention (NACP). The purpose of our study is to understand what Native American women with disabilities and their caregivers think about cancer screenings.

Before we confirm your participation in the study, I need to first complete a brief screening questionnaire with you today. Completing this screener and participating in this study are completely voluntary and you may choose to stop participating at any time.

Date Participant Volunteered: __________

Date Screener Completed: ____________

Site:

- Hopi Cancer Support Services
- Tucson Indian Center

1. How old are you?

_______

[If under age 18 then not eligible]

1. Are you Native American? *Prompt with “or American Indian,” if unsure*

- Yes
- No
- Unsure
  [If yes, then eligible. If no or unsure then, ineligible]

1. Were you born as a boy or a girl? (Check one):

- Boy
- Girl
- Choose not to disclose

[if girl, then eligible. If boy or choose not to disclose, ineligible]

1. Do you identify as having a disability?

- Yes
- No
- Unsure

[If yes, then eligible, skip to #7, and questions 5 and 6 appear on the Demographics survey for woman with IDD. If no or unsure, go to 5.]

1. Do you receive any help from Arizona’s Division of Developmental Disabilities?

- Yes
- No
- Unsure

[If yes, then eligible, skip to question 7, and question 6 appears on the demographics survey for woman with IDD. If no or unsure, go to question 6.]

1. When you were in school, did you ever receive any special education services, require extra support for learning, or feel as if you would have benefitted from support?

- Yes
- No
- Unsure

[If yes, then eligible and go to #7. If no or unsure, ineligible, do not go to question 7]

1. Do you have a legal guardian?

- Yes
- No

[If yes, skip to assent. If no, skip to consent document.]

**Screener form Caregiver**

**Introduction**

Hello my name is ____. I am working on a project with the Partnership for Native American Cancer Prevention (NACP). The purpose of our study is to understand what Native American women with disabilities and their caregivers think about cancer screenings.

Before we confirm your participation in the study, I need to first complete a brief screening questionnaire with you today. Completing this screener and participating in this study are completely voluntary and you may choose to stop participating at any time.

Date Participant Volunteered: __________

Date Screener Completed: ____________

Site:

- Hopi Cancer Support Services
- Tucson Indian Center

1. How old are you?

_______

[If under age 18 then not eligible]

1. Are you a family or friend caregiver for a Native American woman with an intellectual or developmental disability?

- Yes
- No

[If yes, then eligible for study. If no, see next question]

1. Are you a professional support person for a Native American woman with an intellectual or developmental disability?

- Yes
- No

[If yes, then eligible for study. If no, to #2 and #3 ineligible for study.]

**BASELINE – DEMOGRAPHICS (All Participants)**

Participant Role:

- Person with IDD
- Caregiver

Today, I am going to ask you some demographic questions.

1. Which race do you consider yourself? (select all that apply)

- Asian: ______________________
- Black or African-American
- Hispanic or Latinx/o/a or Chicano/a
- Multi-racial
- Native American/American Indian or Alaska Native
- Native Hawaiian or Pacific Islander
- White
- Is there another way you describe yourself?

1. What language is most comfortable for you to speak?

- English
- Hopi
- Spanish
- American Sign Language
- Other (Please specify___________________________)

1. What language is most comfortable for you to read?

- English
- Hopi
- Spanish
- Other (Please specify___________________________)

1. Woman with IDD: Do you use assistive technology to communicate with others?
   *Interviewer may suggest examples*

- No
- Yes. Please describe: ______________________________________________________

Caregiver: Do you use assistive technology to communicate with the woman you

care for?

- No
- Yes. Please describe: ______________________________________________________

1. What is your level of education?

- Less than a High School diploma
- High School Diploma or GED
- Some College
- College Degree
- Some graduate school
- Graduate Degree (e.g. Master’s degree, PhD)
- Not sure

1. What is your current gender identity? (Check one):

- Male

[If person checked “Caregiver” above and “Male” here, skip to the Male Caregiver survey]

- Female

[If person checked “Caregiver” above and “Female” here, skip to the Female Caregiver survey]

- Additional Gender Category, please specify: ______________

[If person checked “Caregiver” above and “Additional Gender Category” here, skip to the Male Caregiver survey]

- Choose not to disclose

[If person checked “Caregiver” above and “Choose not to disclose” here, skip to the Male Caregiver survey]

Caregiver Support Questions:

Please describe for me how much help the woman you support requires with the following activities. Do they need *a lot of help*, *some help*, or *no help* at all?

| **Home Activities** | **A lot of help** | **Some help** | **No help** |
| --- | --- | --- | --- |
| Eating |  |  |  |
| Going to the bathroom |  |  |  |
| Getting dressed |  |  |  |
| Bathing |  |  |  |
| Getting around the house |  |  |  |
| Managing medications |  |  |  |

| **Community Activities** | **A lot of help** | **Some help** | **No help** |
| --- | --- | --- | --- |
| Participating in community activities |  |  |  |
| Getting from place to place |  |  |  |
| Managing money/making purchases |  |  |  |
| Accessing health services |  |  |  |
| If employed, maintaining employment |  |  |  |
| Maintaining their safety |  |  |  |

**Woman with IDD Questionnaire**

*Additional Demographic Questions – Baseline Only*

Where do you live?

Alone

With a partner (a boyfriend/girlfriend, husband/wife)

With a friend

With family (including parents, grandparents, children)

In a group home

Do you have a job?

Yes [if yes, show next question]

No [if no, skip to Part 1]

Do you work:

Full Time (40 hours per week)

Part time (20 or less hours per week)

Where do you work? (e.g. company or agency) [open ended question]

**Women with a disability --**  **BASELINE and FOLLOW-UP (PRE- POST)**

Was caregiver/support person/guardian present for this interview?

- Yes
- No

**PART 1: Mammogram**

*“A mammogram machine is something a doctor uses to check your body. Here is a picture of a mammogram machine. Today I am pretending to go to the doctor’s office and I will be asked to get a mammogram.”*

Picture of mammography Machine

*“Think back to a time that you went to the doctor’s office. Now, I want you to pretend that you are at the doctor’s office with me. I need your help in understanding what the doctor is asking me to do. Thank you for helping me.”*

*“Do your best. If you don’t know the answer it is okay.”*

*“Please make sure you help me on your own, do not have your family or others help you.”*

[Facilitator: If there are questions about the picture, please say that we will talk more about how the mammogram machine works later instead of answering questions.]

*“Now I’m going to ask you what you know about the mammogram machine and how it is used.”*

**Mammogram knowledge:**

1. What parts of the body is the mammogram for
2. Why would I have a mammogram?
3. Would I have to take my clothes off for a mammogram?
4. When I have the mammogram how long will I be in the machine?
5. How often am I supposed to have a mammogram?

*“Thank you for telling me what you know about mammograms. Now I’m going to ask you more questions about your experience with mammograms. “A mammogram is used to screen for breast cancer. A mammogram is a test often given to women over 40 years old.”*

**Mammogram Prior Screening History:**

1. Have you ever had a mammogram?

- Yes
- No
- I don’t know

[If no or I don’t know, skip to self-efficacy questions.]

1. During the past 3 years, have you had a mammogram?

- No
- Yes, once
- Yes, more than once
- Don’t know

**Mammogram Self-efficacy:**

*“Imagine that this ladder is a way of picturing your confidence, or how* ***sure*** *you are that you can do something. The top of the ladder means that you are very sure, and the bottom means you are not sure at all. For these next questions, what place on the ladder (or number between 0 and 10) matches how sure you feel.”*

*
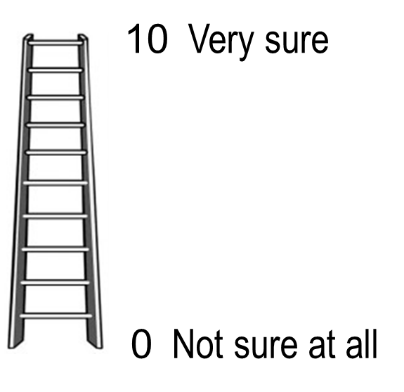
*

1. How sure are you that you can decide whether or not to get a mammogram?
   I------------------------------------------------------------------------------------------I
   0 10

Not sure at all Very sure

1. How sure are you that you can get a mammogram?

I------------------------------------------------------------------------------------------I
0 10

Not sure at all Very sure

1. How sure are you that you can get a mammogram, even if you are nervous about it?
   I------------------------------------------------------------------------------------------I
   0 10

Not sure at all Very sure

1. How sure are you that you can do what is needed to prepare for a mammogram?
   I------------------------------------------------------------------------------------------I
   0 10

Not sure at all Very sure

*“Now I’m going to ask you about your plans to get a mammogram.”*

**Mammogram Expectations:**

1. I plan to get a mammogram.

I------------------------------------------------------------------------------------------I
0 10
Not at all planning to get a mammogram Definitely planning

1. I plan to discuss getting a mammogram with my doctor.
   I------------------------------------------------------------------------------------------I
   0 10
   Not at all planning to get a mammogram Definitely planning
2. I plan to discuss getting a mammogram with people who support me (for example: family member, friend, staff, helper, direct support professional).

I------------------------------------------------------------------------------------------I
0 10
Not at all planning to get a mammogram Definitely planning

*“Thank you. Now I am going to ask you questions about getting a Pap test.”*

**PART 2: Pap testing**

*“Here is a picture of an exam table I would lay down on to get a Pap test. Today I am pretending to go to the doctor’s office and will be asked to get a Pap test.”*

Picture of exam room.

*“Now I’m going to ask you what you know about getting a Pap test.”*

**Pap test knowledge:**

1. What body part is a Pap test for?
2. Why would I have a Pap test?
3. Would I have to take my clothes for a Pap test?
4. When I have my Pap test, how long will it take?
5. How often should I have a Pap test?

*“Thank you for telling me what you know about getting a Pap test. Now I’m going to ask you more questions about your experience with getting a Pap test.* *A Pap test is used to screen for cervical cancer. A Pap test is a test often given to women over 21 years old.”*

**Pap test Prior Screening History:**

1. Have you ever had a Pap test?

- Yes
- No
- I don’t know

[If no or I don’t know, skip to self-efficacy questions.]

1. During the past 3 years, have you had a pap test?

- No,
- Yes, once,
- Yes, more than once
- Don’t know

*“Imagine that this ladder is a way of picturing your confidence, or how* ***sure*** *you are that you can do something. The top of the ladder means that you are very sure, and the bottom means you are not sure at all. For these next questions, what place on the ladder (or number between 0 and 10) matches how sure you feel.”*

*
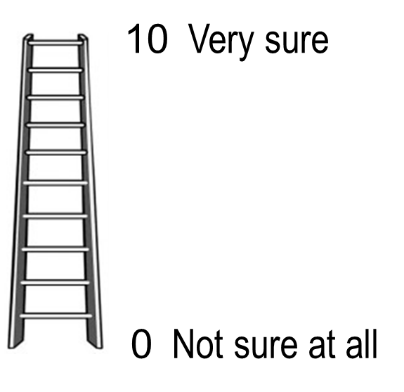
*

**Pap test Self-efficacy:**

1. How sure are you that you can decide whether or not to get a Pap test?

I------------------------------------------------------------------------------------------I
0 10

Not sure at all Very sure

1. How sure are you that you can get a Pap test?

I------------------------------------------------------------------------------------------I
0 10

Not sure at all Very sure

1. How sure are you that you can get a Pap test even if you are nervous about it?

I------------------------------------------------------------------------------------------I
0 10

Not sure at all Very sure

How sure are you that you can do what is needed to prepare for a Pap test?

1. I------------------------------------------------------------------------------------------I
   0 10

Not sure at all Very sure

*“Now I’m going to ask you about your plans to get a Pap test.”*

**Pap test Expectations:**

1. I plan to get a Pap test.

I------------------------------------------------------------------------------------------I
0 10
Not at all planning to get a Pap test Definitely planning

1. I plan to discuss getting a Pap test with my doctor:
   I------------------------------------------------------------------------------------------I
   0 10
   Not at all planning to get a Pap test Definitely planning
2. I plan to discuss getting a Pap test with people who support me (for example: family member, friend, staff, helper, direct support professional).

I------------------------------------------------------------------------------------------I
0 10
Not at all planning to get a Pap test Definitely planning

*“Thank you for completing this interview. Now I would like your support person to complete an interview”*

[Facilitator: If caregiver is a guardian and already present, proceed. If caregiver not present, then ask the woman to now invite the caregiver to join you for the interview]

**Female Caregiver Questionnaire – BASELINE and FOLLOW-UP (PRE- POST)**

**PART 1: Mammogram**

*“A mammogram machine is something a doctor uses to check your body. Here is a picture of a mammogram machine. Today I am pretending to go to the doctor’s office and will be asked to get a mammogram.”*

Picture of mammography Machine

*“Think back to a time that you went to the doctor’s office. Now, I want you to pretend that you are at the doctor’s office with me. I need your help in understanding what the doctor is asking me to do. Thank you for helping me.”*

*“Do your best. If you don’t know the answer it is okay.”*

*“Please make sure you help me on your own, do not have your family or others help you.”*

[Facilitator: If there are questions about the picture, please say that we will talk more about how the mammogram machine works later instead of answering questions.]

*“Now I’m going to ask you what you know about the mammogram machine and how it is used.”*

**Mammogram knowledge:**

1. What parts of the body is the mammogram for?
2. Why would I have a mammogram?
3. Would I have to take my clothes off for a mammogram?
4. When I have the mammogram how long will I be in the machine?
5. How often am I supposed to have a mammogram?

*“Thank you for telling me what you know about mammogram. Now I’m going to ask you more questions about your experience with mammograms. A mammogram is a test often given to women over 40 years old.”*

**Mammogram Prior Screening History:**

1. Have you ever had a mammogram?

- Yes
- No
- I don’t know

[If no or I don’t know, skip to self-efficacy questions.]

1. During the past 3 years, have you had a mammogram?

- No
- Yes, once
- Yes, more than once
- Don’t know

**Self-efficacy questions**

*“A mammogram is used to screen for breast cancer. Now I’m going to ask you about your plans to get a mammogram and support the woman with a disability you care for in her plans to get a mammogram. Can you tell me how you refer to the woman you care for?”* [answer here]

*“Imagine that this ladder is a way of picturing your confidence, or how* ***sure*** *you are that you can do something. The top of the ladder means that you are very sure, and the bottom means you are not sure at all. For these next questions, what place on the ladder (or number between 0 and 10) matches how sure you feel.”*

*
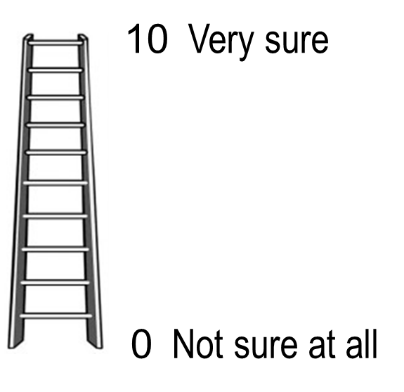
*

**Mammogram Self-efficacy:**

1. How sure are you that you can decide whether or not to get a mammogram?

I------------------------------------------------------------------------------------------I
0 10

Not sure at all Very sure

1. How sure are you that you can get a mammogram?

I------------------------------------------------------------------------------------------I
0 10

Not sure at all Very sure

1. How sure are you that you can get a mammogram, even if you are nervous about it?

I------------------------------------------------------------------------------------------I
0 10

Not sure at all Very sure

1. How sure are you that you can do what is needed to prepare for a mammogram?

I------------------------------------------------------------------------------------------I
0 10

Not sure at all Very sure

**Mammogram Support Self-efficacy:**

1. How sure are you that you can support [insert woman with disability’s name here] to get a mammogram?

I------------------------------------------------------------------------------------------I
0 10

Not sure at all Very sure

1. How sure are you that you can support [insert woman with a disability’s name here] in deciding whether or not to get a mammogram?

I------------------------------------------------------------------------------------------I
0 10

Not sure at all Very sure

1. How sure are you that you can support [insert woman with a disability’s name here] in getting a mammogram, even if she is nervous about it?

I------------------------------------------------------------------------------------------I
0 10

Not sure at all Very sure

**Mammogram Support Expectations**:

1. I plan to assist [insert woman with a disability’s name here] in getting a mammogram:
   l-----------------------------------------------------------------------------------------------I

0 10

Not at all planning to assist Definitely planning

1. I plan to support [insert woman with a disability’s name here] to discuss getting a mammogram with the her doctor:
   l-----------------------------------------------------------------------------------------------I

0 10

Not at all planning to assist Definitely planning

**Mammogram Expectations**:

1. I plan to get a mammogram.

I------------------------------------------------------------------------------------------I
0 10
Not at all planning to get a mammogram Definitely planning

1. I plan to discuss getting a mammogram with my doctor.
   I------------------------------------------------------------------------------------------I
   0 10
   Not at all planning to get a mammogram Definitely planning
2. I plan to assist [insert woman with a disability’s name here] in getting a mammogram.
   l-----------------------------------------------------------------------------------------------I
3. 10

Not at all planning to assist Definitely planning

1. I plan to support [insert woman with a disability’s name here] to discuss getting a mammogram with her doctor.

l-----------------------------------------------------------------------------------------------I

0 10

Not at all planning to assist Definitely planning

*“Thank you for telling me what you know about getting a mammogram.*

*Now I am going to ask you questions about getting a Pap test.”*

**PART 2: Pap test**

*“Here is a picture of an exam table I would lay down on to get a Pap test. Today I am pretending to go to the doctor’s office and will be asked to get a Pap test.”*

Picture of exam room.

*“Now I’m going to ask you what you know about getting a Pap test.”*

**Pap test knowledge:**

1. What body part is a Pap test for?
2. Why would I have a Pap test?
3. Would I have to take my clothes for a Pap test?
4. When I have my Pap test, how long will it take?
5. How often should I have a Pap test?

*“Thank you for telling me what you know about getting a Pap test. Now I’m going to ask you more questions about your experience with getting a Pap test. A Pap test is a test often given to women over 21 years old.”*

**Pap test Prior Screening History:**

1. Have you ever had a Pap test?

- Yes
- No
- I don’t know

[If no or I don’t know, skip to self-efficacy questions.]

1. During the past 3 years, have you had a pap test?

- No
- Yes, once
- Yes, more than once
- Don’t know

*“A Pap test is used to screen for cervical cancer. Now I’m going to ask you about your plans to get a Pap test and your plans to support the woman with a disability you care for in her plans to get a Pap test.”*

*“Imagine that this ladder is a way of picturing your confidence, or how* ***sure*** *you are that you can do something. The top of the ladder means that you are very sure, and the bottom means you are not sure at all. For these next questions, what place on the ladder (or number between 0 and 10) matches how sure you feel.”*

*
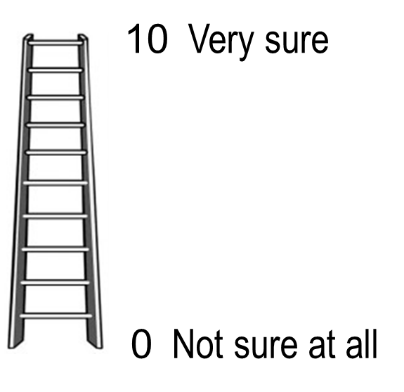
*

**Pap test Self-efficacy:**

1. How sure are you that you can decide whether or not to get a Pap test?

I------------------------------------------------------------------------------------------I
0 10

Not sure at all Very sure

1. How sure are you that you can get a Pap test?

I------------------------------------------------------------------------------------------I
0 10

Not sure at all Very sure

1. How sure are you that you can get a Pap test, even if you are nervous about it?

I------------------------------------------------------------------------------------------I
0 10

Not sure at all Very sure

1. How sure are you that you can do what is needed to prepare for a Pap test?

I------------------------------------------------------------------------------------------I
0 10

Not sure at all Very sure

**Pap-Test** **Support Self-efficacy:**

1. How sure are you that you can support [insert woman with a disability’s name here] to get a Pap test?

I------------------------------------------------------------------------------------------I
0 10

Not sure at all Very sure

1. How sure are you that you can support [insert woman with a disability’s name here] in deciding whether or not to get a Pap test?

I------------------------------------------------------------------------------------------I
0 10

Not sure at all Very sure

1. How sure are you that you can support [insert woman with a disability’s name here] in getting a Pap test, even if she is nervous about it?

I------------------------------------------------------------------------------------------I
0 10

Not sure at all Very sure

**Pap test Expectations**:

1. I plan to get a Pap test.

I------------------------------------------------------------------------------------------I
0 10
Not at all planning to get a Pap test Definitely planning

1. I plan to discuss getting a Pap test with my doctor.
   I------------------------------------------------------------------------------------------I
   0 10
   Not at all planning to get a Pap test Definitely planning
2. I plan to assist [insert woman with a disability’s name here] in getting a Pap test.
   l-----------------------------------------------------------------------------------------------I

0 10

Not at all planning to assist Definitely planning

1. I plan to support [insert woman with a disability’s name here] to discuss getting a Pap test with her doctor.

l-----------------------------------------------------------------------------------------------I

0 10

Not at all planning to assist Definitely planning

*“Thank you for completing this interview.”*

**Male Caregiver Questionnaire - BASELINE and FOLLOW-UP (PRE- POST)**

**PART 1: Mammogram**

*“A mammogram machine is something a doctor uses to check your body. Here is a picture of a mammogram machine. Today I am pretending to go to the doctor’s office and will be asked to get a mammogram.”*

Picture of mammography Machine

*“Think back to a time that you went to the doctor’s office. Now, I want you to pretend that you are at the doctor’s office with me. I need your help in understanding what the doctor is asking me to do. Thank you for helping me.”*

*“Do your best. If you don’t know the answer it is okay.”*

*“Please make sure you help me on your own, do not have your family or others help you.”*

[Facilitator: If there are questions about the picture, please say that we will talk more about how the mammogram machine works later instead of answering questions.]

*“Now I’m going to ask you what you know about the mammogram machine and how it is used.”*

**Mammogram knowledge:**

1. What parts of the body is the mammogram for?
2. Why would I have a mammogram?
3. Would I have to take my clothes off for a mammogram?
4. When I have the mammogram how long will I be in the machine?
5. How often am I supposed to have a mammogram?

*“Thank you for telling me what you know about mammograms. Now I’m going to ask you more questions about your experience in supporting the woman with a disability to get her mammogram.”*

*“Imagine that this ladder is a way of picturing your confidence, or how* ***sure*** *you are that you can do something. The top of the ladder means that you are very sure, and the bottom means you are not sure at all. For these next questions, what place on the ladder (or number between 0 and 10) matches how sure you feel.”*

*
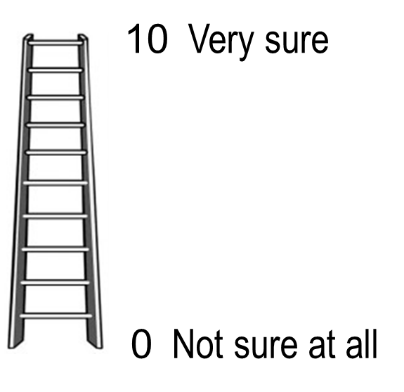
*

**Mammogram Support Self-efficacy:**

1. How sure are you that you can support [insert woman with disability’s name here] to get a mammogram?

I------------------------------------------------------------------------------------------I
0 10

Not sure at all Very sure

1. How sure are you that you can support [insert woman with a disability’s name here] in deciding whether or not to get a mammogram?

I------------------------------------------------------------------------------------------I
0 10

Not sure at all Very sure

1. How sure are you that you can support [insert woman with a disability’s name here] in getting a mammogram, even if she is nervous about it?

I------------------------------------------------------------------------------------------I
0 10

Not sure at all Very sure

**Mammogram Support Expectations**:

1. I plan to assist [insert woman with a disability’s name here] in getting a mammogram:
   l-----------------------------------------------------------------------------------------------I

0 10

Not at all planning to assist Definitely planning

1. I plan to support [insert woman with a disability’s name here] to discuss getting a mammogram with the her doctor:
   l-----------------------------------------------------------------------------------------------I

0 10

Not at all planning to assist Definitely planning

*“Thank you. Now I am going to ask you questions about getting a Pap test.”*

**PART 2: Pap test**

*“Here is a picture of an exam table I would lay down on to get a Pap test. Today I am pretending to go to the doctor’s office and will be asked to get a Pap test.”*

Picture of exam room.

*“Now I’m going to ask you what you know about getting a Pap test.”*

**Pap test knowledge:**

1. What body part is a Pap test for?
2. Why would I have a Pap test?
3. Would I have to take my clothes for a Pap test?
4. When I have my Pap test, how long will it take?
5. How often should I have a Pap test?

*“Thank you for telling me what you know about getting a Pap test.”*

*“Imagine that this ladder is a way of picturing your confidence, or how* ***sure*** *you are that you can do something. The top of the ladder means that you are very sure, and the bottom means you are not sure at all. For these next questions, what place on the ladder (or number between 0 and 10) matches how sure you feel.”*

*
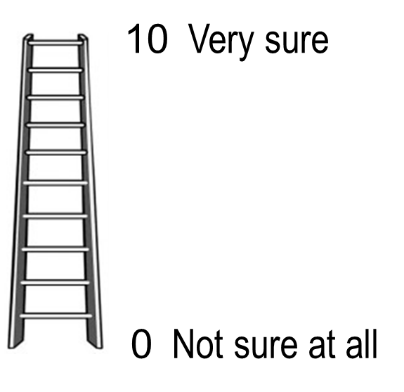
*

**Pap-Test** **Support Self-efficacy:**

1. How sure are you that you can support [insert woman with a disability’s name here] to get a Pap test?

I------------------------------------------------------------------------------------------I
0 10

Not sure at all Very sure

1. How sure are you that you can support [insert woman with a disability’s name here] in deciding whether or not to get a Pap test?

I------------------------------------------------------------------------------------------I
0 10

Not sure at all Very sure

1. How sure are you that you can support [insert woman with a disability’s name here] in getting a Pap test, even if she is nervous about it?

I------------------------------------------------------------------------------------------I
0 10

Not sure at all Very sure

**Pap test Expectations**:

1. I plan to get a Pap test.

I------------------------------------------------------------------------------------------I
0 10
Not at all planning to get a Pap test Definitely planning

1. I plan to discuss getting a Pap test with my doctor.
   I------------------------------------------------------------------------------------------I
   0 10
   Not at all planning to get a Pap test Definitely planning
2. I plan to assist [insert woman with a disability’s name here] in getting a Pap test.
   l-----------------------------------------------------------------------------------------------I

0 10

Not at all planning to assist Definitely planning

1. I plan to support [insert woman with a disability’s name here] to discuss getting a Pap test with her doctor.

l-----------------------------------------------------------------------------------------------I

0 10

Not at all planning to assist Definitely planning

*“Thank you for completing this interview.”*

**HEALTH EDUCATOR SESSION FEASIBILITY AND ACCEPTABILITY DATA COLLECTION**

Session 1 – 6 should have their own forms with the following data:

1. Session date
2. Session start time
3. Session end time
4. Participants have the toolkit.

- Yes
- No

1. Program delivery method:

- Zoom
- Phone call
- Facetime
- Facebook messenger
- Whatsapp
- Other

1. Were you able to complete all parts of the session?

- Yes
- No
  1. If not, please list the content you did not complete.

1. On a scale of 1 to 5 with 1 being not at all effective and 5 being very effective, give your overall impression of the session.

Not all effective 1 2 3 4 5 Very effective

- 1. Please explain your effectiveness rating: _________________________

1. The woman with IDD was (check one from each pair):

- Engaged
- Not engaged
- Calm
- Anxious
- Enthusiastic
- Unenthusiastic
- Expressive
- Not expressive
- Talkative
- Quiet
- Other, please describe_______________________________

1. The caregiver (check one from each pair):

- Was present
- Was not present *(skip to 10)*
- Participated
- Did not participate *(skip to 10)*
- Was helpful
- Was not helpful *(explain or give examples in either case)*

Please share any other thoughts about the caregiver’s involvement:

 ______________________________________________________

1. In general, how would you rate **communication** during the session?

Not all effective 1 2 3 4 5 Very effective

10a) Please describe any specific issues with **communication** (required for ratings less than 4 or 5) _________________________________

1. In general, how would you rate **technology** used during the session?

Never effective 1 2 3 4 5 Consistently effective

11a) Please describe any specific issues with **technology** (required for ratings less than 4 or 5) ___________________________________________

1. Were there distractions or interruptions?

- Yes
- No

If *yes*, please describe and offer suggestions for minimizing distractions/interruptions.

1. Please describe any other issues that might have influenced the session.

14) Educator Notes and General comments about the session

Texted questions to all participants:

1. Did you learn something new in My Health My Choice today?

- Yes
- No
- Not sure

1. Overall, did you enjoy My Health My Choice today?

 Yes

 No

 Not sure

1. Please share your thoughts about today’s My Health My Choice session.
